# Brain-Specific Increase in Leukotriene Signaling Accompanies Chronic Neuroinflammation and Cognitive Impairment in a Model of Gulf War Illness

**DOI:** 10.3389/fimmu.2022.853000

**Published:** 2022-04-29

**Authors:** Sahithi Attaluri, Raghavendra Upadhya, Maheedhar Kodali, Leelavathi N. Madhu, Dinesh Upadhya, Bing Shuai, Ashok K. Shetty

**Affiliations:** Institute for Regenerative Medicine, Department of Molecular and Cellular Medicine, College of Medicine, Texas A&M University Health Science Center, College Station, TX, United States

**Keywords:** cysteinyl leukotrienes, gulf war illness (GWI), gulf war-related chemicals, cytokines, 5-lipoxygenase, neuroinflammation, leukotriene signaling, blood-brain barrier

## Abstract

Persistent cognitive impairment is a primary central nervous system-related symptom in veterans afflicted with chronic Gulf War Illness (GWI). Previous studies in a rat model have revealed that cognitive dysfunction in chronic GWI is associated with neuroinflammation, typified by astrocyte hypertrophy, activated microglia, and enhanced proinflammatory cytokine levels. Studies in a mouse model of GWI have also shown upregulation of several phospholipids that serve as reservoirs of arachidonic acid, a precursor of leukotrienes (LTs). However, it is unknown whether altered LT signaling is a component of chronic neuroinflammatory conditions in GWI. Therefore, this study investigated changes in LT signaling in the brain of rats displaying significant cognitive impairments six months after exposure to GWI-related chemicals and moderate stress. The concentration of cysteinyl LTs (CysLTs), LTB4, and 5-Lipoxygenase (5-LOX), the synthesizing enzyme of LTs, were evaluated. CysLT and LTB4 concentrations were elevated in the hippocampus and the cerebral cortex, along with enhanced 5-LOX expression in neurons and microglia. Such changes were also associated with increased proinflammatory cytokine levels in the hippocampus and the cerebral cortex. Enhanced CysLT and LTB4 levels in the brain could also be gleaned from their concentrations in brain-derived extracellular vesicles in the circulating blood. The circulating blood in GWI rats displayed elevated proinflammatory cytokines with no alterations in CysLT and LTB4 concentrations. The results provide new evidence that a brain-specific increase in LT signaling is another adverse alteration that potentially contributes to the maintenance of chronic neuroinflammation in GWI. Therefore, drugs capable of modulating LT signaling may reduce neuroinflammation and improve cognitive function in GWI. Additional findings demonstrate that altered LT levels in the brain could be tracked efficiently by analyzing brain-derived EVs in the circulating blood.

## Introduction

Cognitive and mood impairments associated with neuroinflammation and systemic inflammation are among the most noticeable and enduring health issues in GWI (1–7). Studies have implied that GWI in a vast majority of veterans is an aftereffect of exposure to several chemicals and/or their interaction with war-related stress (5, 8, 9). The chemicals include the nerve gas prophylactic drug pyridostigmine bromide (PB), the mosquito repellant DEET, the insecticide permethrin (PER), the pesticide chlorpyrifos, the organophosphate sarin, and depleted uranium (2, 5, 7, 10). PB was consumed as a prophylactic measure against the possible nerve agent attack by the enemy (2). The mosquito repellants, insecticides, and pesticides were used as sprays on the skin, uniforms, or tents as a countermeasure against the infectious diseases transmitted by insects and ticks in the desert (2, 5). A series of studies in a rat model demonstrated that oral administration of PB, along with a dermal application of low doses of DEET and PER for four weeks with or without mild to moderate stress, results in chronic GWI-like symptoms with multiple pathological changes in the brain have corroborated the chemical exposure hypothesis of GWI (11–19). Additional animal studies utilizing exposure to sarin surrogate diisopropyl fluorophosphate, chlorpyrifos, or various combinations of GWI-related chemicals have also validated the chemical exposure hypothesis of GWI (20–31).

Notably, studies in both rat and mouse models of GWI have demonstrated that persistent neuroinflammation continually accompanies cognitive and/or mood dysfunction in GWI (9, 11, 16–19, 32, 33). Neuroinflammation was apparent from the persistence of proinflammatory M1 microglia with activation of NOD-, LRR- and pyrin domain-containing protein 3 (NLRP3) inflammasomes, hypertrophy of astrocytes, and increased concentration of proinflammatory cytokines (18, 19). Moreover, these changes were associated with the translocation of high mobility group box-1 (HMGB1) into the neuronal cytoplasm, its leakage into the extracellular space, and complement activation in the brain (16). Nonetheless, precise mechanisms sustaining microglial activation and reactive astrocytes after exposure to GWI-related chemicals are unknown.

A few studies have reported upregulation of phosphatidylcholine (PC) in the brain of mice with chronic GWI-like symptoms (34, 35). Increased PC levels are also seen in the circulating blood of veterans with chronic GWI (36, 37). PC is the primary reservoir for ω-6 arachidonic acid (AA), which is a precursor in the biosynthesis of proinflammatory leukotrienes (LTs). Therefore, it is plausible that increased LT signaling in the brain is one of the mechanisms sustaining chronic neuroinflammation. LTs, chemical messengers that signal from immune cells to other cell types, comprise LTB4, and cysteinyl LTs (CysLTs) LTC4, LTD4, and LTE4. LTB4 promotes the production of cytokines by immune cells (38). The CysLTs act on receptors CysLTR1 and CysLTR2 expressed in target cells, including microvascular endothelial cells, microglia, astrocytes, and neurons in the brain (39, 40). While the healthy brain produces minimal LTs, an injury or disease enhances LT synthesis in the brain. For example, both LTB4 and CysLTs are elevated after traumatic brain injury (41), and in cerebral ischemia (42), epilepsy (43), and Alzheimer’s disease (44). Increased LTB4 can promote neurodegeneration (45), whereas elevated CysLTs can induce the disruption of the blood-brain barrier (BBB) (46).

Astrocytes and microglia are key players in inflammation associated with neurodegenerative diseases, including GWI. Since CysLTs and CysLT receptors (CysLTRs) regulate their functions (46–48), increased LT signaling can maintain detrimental chronic neuroinflammation typified by activated microglia and astrocyte hypertrophy with leaky BBB. However, it is unknown whether altered LT signaling is a component of chronic neuroinflammatory conditions in GWI. Therefore, we investigated changes in LT signaling in the brain of rats displaying significant cognitive impairments six months after exposure to GWI-related chemicals and moderate stress. We measured CysLT and LTB4 concentrations in the hippocampus and the cerebral cortex, 5-LOX expression in neurons, microglia and astrocytes, and proinflammatory cytokines in the hippocampus and the cerebral cortex. We also investigated whether CysLT and LTB4 levels in the brain could be tracked noninvasively by analyzing brain-derived extracellular vesicles (EVs) in the circulating blood. We also measured LTs and proinflammatory cytokines in the circulating blood to ascertain whether increased LT signaling is a global or a brain-specific alteration in GWI. Our results suggest that chronic neuroinflammation and cognitive dysfunction in GWI are linked with a brain-specific increase in LT signaling, and such change could be tracked by analyzing brain-derived EVs in the blood.

## Materials and Methods

### Animals

Approximately two-month-old male Sprague Dawley rats weighing 250-280 gms (n=27) were procured from Harlan (Indianapolis, IN, USA). The animals were housed in the vivarium at 18-23 degrees C with 40-60% humidity, ad libitum access to food and water, and 12 hour light/dark cycles. After two weeks of familiarization, the animals were randomly assigned to either the naive control group (n=14) or the GWI group (n=13). The Texas A&M Health Science Center College of Medicine’s institutional animal care and use committee authorized all animal experiments performed in this study. We focused on male rats to study GWI pathogenesis in this study because most of the service personnel who participated in the first Gulf War were males (7).

### Exposure to GWI-Related Chemicals and Induction of Restraint Stress

Daily applications of GWI-related chemicals and induction of restraint stress were conducted for 28 days, as described in our previous reports (17–19). Briefly, the animals in the GWI group received PB orally (2 mg/Kg in 500 µl of sterile water, Sigma, St. Louis, MO, USA) and dermal applications of DEET (60 mg/Kg, Chem Service Inc, West Chester, PA, USA) and PER (0.2 mg/Kg, Chem Service Inc) dissolved in 70% alcohol. 15-minute restraint stress was induced daily using a rat restrainer (Stoelting Research Instruments, Wood Dale, IL, USA), as described in our previous study (49). Both rats exposed to chemicals and stress (i.e., GWI rats) and age-matched naïve control rats were maintained in the vivarium with ad libitum access to food and water.

### Time-Line of Behavioral Tests

Six months after exposure to GWI-related chemicals and stress, the GWI rats were tested for recognition memory function using a novel object recognition test (NORT) and temporal pattern processing ability using a temporal pattern processing test (TPPT). The age-matched naïve control animals also underwent similar behavioral testing. The purpose of behavioral tests was to confirm that GWI rats used in the study display recognition memory and temporal pattern processing impairments at the time of biochemical and histological studies on their brain tissues.

### Investigation of Recognition Memory Function in GWI Rats

The rats were tested for recognition memory function using a NORT (n=13-14/group), as detailed in our previous reports (12, 15, 16, 18, 50). In brief, each animal sequentially explored an empty open field box for 5 min (trial 1, T1), and two identical objects placed obliquely in the open field apparatus for 5 min (T2). After an inter-trial interval of 30 minutes, the animal was allowed to explore one of the objects from T2 (i.e., the familiar object) and a novel object. The behavior of animals in T2 and T3 was video-recorded and analyzed using AnyMaze software (Stoelting Co., IL, USA). Only animals that explored objects for 16 seconds in T2 and 8 seconds in T3 were included for data analysis. The percentages of exploration times spent with the novel object vis-`a-vis the familiar object were statistically compared within each group. Also, the novel object discrimination index was calculated and compared across groups using the formula, the time spent with the novel object/the total object exploration time (TOET) ×100. Additional measures from T2 such as TOETs, total distances traveled, and velocities of the movement were also compared between the two groups.

### Examination of Temporal Pattern Processing Ability in GWI Rats

The rats were probed for their ability for temporal pattern processing using a spontaneous object recognition paradigm (n=12/group). Following familiarization with the open field apparatus for 8 minutes (T1), each animal successively explored three distinct pairs of identical objects (T2-T4, 8 minutes each) with an inter-trial interval of 60 minutes. In T5, the animal was placed in the open field for 8 minutes with a recently explored object (i.e., one object from T4) and a previously explored object (i.e., one object from T2). The propensity of the animal to prefer the previously explored object over the recently explored object was analyzed. Any-maze video tracking device tracked the activity of each animal from T2-T5. The amounts of time spent with the previously explored and recently explored objects in T5, previously explored object discrimination index, and TOETs in T2-T4 were compared across groups. Since the validity of the test is contingent on a thorough examination of objects in T2-T4, only animals that examined objects for 20 seconds in T2-T4 and 8 seconds in T5 were included in the data analysis.

### Brain Tissue Harvesting for Biochemical and Immunofluorescence Studies

Approximately 9.5 months after exposure to GWI-related chemicals and stress, subgroups of rats from each group (n=6-7 each group) were deeply anesthetized, the chest cavity was opened, the blood was collected from the left ventricle of the heart and euthanized by decapitation (14). These brains were quickly removed and snap-frozen with dry ice before being stored in a -80°C freezer for future biochemical experiments. After thawing brain tissues to room temperature, the hippocampus and cerebral cortex were micro-dissected and homogenized individually through sonication in a tissue extraction reagent (Invitrogen, Waltham, MA) containing protease inhibitor (Sigma Aldrich, 1:100 dilution) for 15–20 seconds at 4°C. The resulting solution was centrifuged for 10 min at 15000 g, and the supernatant was aliquoted and stored at −80°C until further use. The lysates were used to measure the concentration of CysLTs, LTBs, and inflammatory markers. The serum was collected from the blood and stored at -80°C, employing standard techniques. Additional animals (n=6-7/group) were deeply anesthetized, perfused through the heart with 4% paraformaldehyde, the fixed brains were removed and processed for cryostat sectioning, as described in our previous reports (51–54). Thirty-micrometer thick brain tissue sections were employed for immunofluorescence studies.

### Analysis of 5-LOX Expression in Neurons, Microglia, and Astrocytes

The brain tissue sections were washed thoroughly in phosphate buffer, treated with 10% normal donkey serum for 30 minutes, and then incubated for 24 hours with a cocktail of primary antibodies against NeuN (rabbit anti-NeuN, 1:1000; EMD Millipore, Burlington, MA, USA) and 5-LOX (mouse anti-5-LOX, 1:500; BD Biosciences, San Jose, Ca, USA) (55), IBA-1 (goat anti-IBA-1, 1:1000; Abcam, Waltham, MA, USA) and 5-LOX, or glial fibrillary acidic protein (rabbit anti-GFAP, 1:2000; Dako, Santa Carla, CA, USA) and 5-LOX. The primary antibodies against NeuN and IBA-1 employed in this study have been validated in our previous reports (11, 15–17). The 5-LOX antibody employed in this study has been used successfully to demonstrate 5-LOX expression in the hippocampus (55). Following washing, the sections were treated with a cocktail of anti-mouse IgG conjugated to Alexa Fluor 594 and anti-rabbit IgG conjugated to Alexa Fluor 488 (1:200, Invitrogen, Waltham, MA, USA) for 60 minutes. The sections were washed, mounted on subbed slides with the slow fade-antifade medium. The percentages of NeuN+ cells expressing 5-LOX and IBA-1+ cells expressing 5-LOX in different hippocampal subfields and the cerebral cortex were determined using Z- section analysis in a confocal microscope.

### Measurement of 5-LOX, LTB4, and CysLTs in Hippocampal and Cerebral Cortical Tissues

LT synthesis involves the oxidation of AA by 5-lipoxygenase (5-LOX) (56–58). AA is released from the cell membrane phospholipids by phospholipase 2, which is converted into LTA4 by 5-LOX (59). LTA4 hydrolase converts LTA4 into LTB4, and LTC4 synthase transforms LTA4 into LTC4 by conjugating with glutathione. ATP-binding cassette transporters export both LTB4 and LTC4 into the extracellular space, where LTC4 is converted into LTD4 through the deletion of glutamate by γ-glutamyl transferase. Removal of glycine by dipeptidase yields LTE4 from LTD4. In this study, to ascertain changes in LTs in the brain, we measured 5-LOX, LTB4, and CysLTs (LTC4, LTD4, LTE4) in hippocampal and cerebral cortical tissue lysates from naive and GWI rats. We employed a 5-LOX competitive immunoassay kit from Novus Biologicals (CO, USA) and LTB4 and CysLT competitive immunoassay kits from Enzo life sciences (Farmingdale, NY, USA). We closely followed the manufacturer’s instructions for these assays. 5-LOX, LTB4, and CysLTs levels were normalized to total protein in lysates.

### Isolation and Characterization of Brain-Derived EVs From the Serum

The EVs in the serum were first collected using Exoquick precipitation reagent (System Biosciences, Palo Alto, CA, USA), and then neuron-derived EVs (NDEVs) and astrocyte-derived EVs (ADEVs) were isolated using methods detailed in our previous report (16). Isolation of NDEVs utilized a mouse anti-human L1CAM (CD171) biotinylated antibody (eBiosciences, San Diego, CA, USA) and streptavidin with ultra-link resin (Thermo Fischer Scientific, Waltham, MA, USA). The separation of ADEVs employed a mouse anti-human glutamine aspartate transporter (ACSA-1) biotinylated antibody (Miltenyi, San Diego, CA, USA) and streptavidin with ultra-link resin. The concentration of CD81 was measured from NDEVs and ADEVs, as described in our earlier study (16). Then, the concentration of LTB4 and CysLTs were measured from both NDEVs and ADEVs, using competitive ELISA kits described earlier. The concentration of LTB4 and CysLTs were normalized to CD81.

### Measurement of Proinflammatory Cytokines in the Hippocampus, Cerebral Cortex and the Serum

We performed double antibody sandwich ELISAs to measure several proinflammatory proteins. These include the tumor necrosis factor-alpha (TNF-α; R&D Systems, MN, USA), interleukin-1 beta (IL-1β; R&D Systems, MN, USA), macrophage inflammatory protein -1 alpha (MIP-1α; Signosis, Santa Clara, CA, USA), and IL-6 (R&D Systems, MN, USA) in hippocampal and cerebral cortical tissue lysates, as described in our previous studies (18, 19, 60). As described in our previous study, we employed a very sensitive rat cytokine array to measure proinflammatory markers in the serum, as per manufacturer instructions (Signosis, Santa Clara, CA, USA) (14). The levels of different proinflammatory cytokines were then compared between GWI rats and naive control rats.

### Statistical Analyses

The sample sizes for behavioral and histological studies were determined through power analysis using the G*Power software. Such analysis using effect size values observed in our previous studies and alpha at 0.05 suggested that to obtain a power of 0.8, data from 10 animals/group are needed for neurobehavioral studies, and 5-6 animals/group are needed for histological studies. A two-tailed, unpaired Student’s *t*-test in the Prism software was employed to compare two data columns. The Mann-Whitney U-test was employed when standard deviations between groups were statistically significant. The values in the bar charts are Mean ± S.E.M., and p<0.05 was considered statistically significant.

## Results

### Recognition Memory Function Was Impaired in GWI Rats

**Figure 1:**
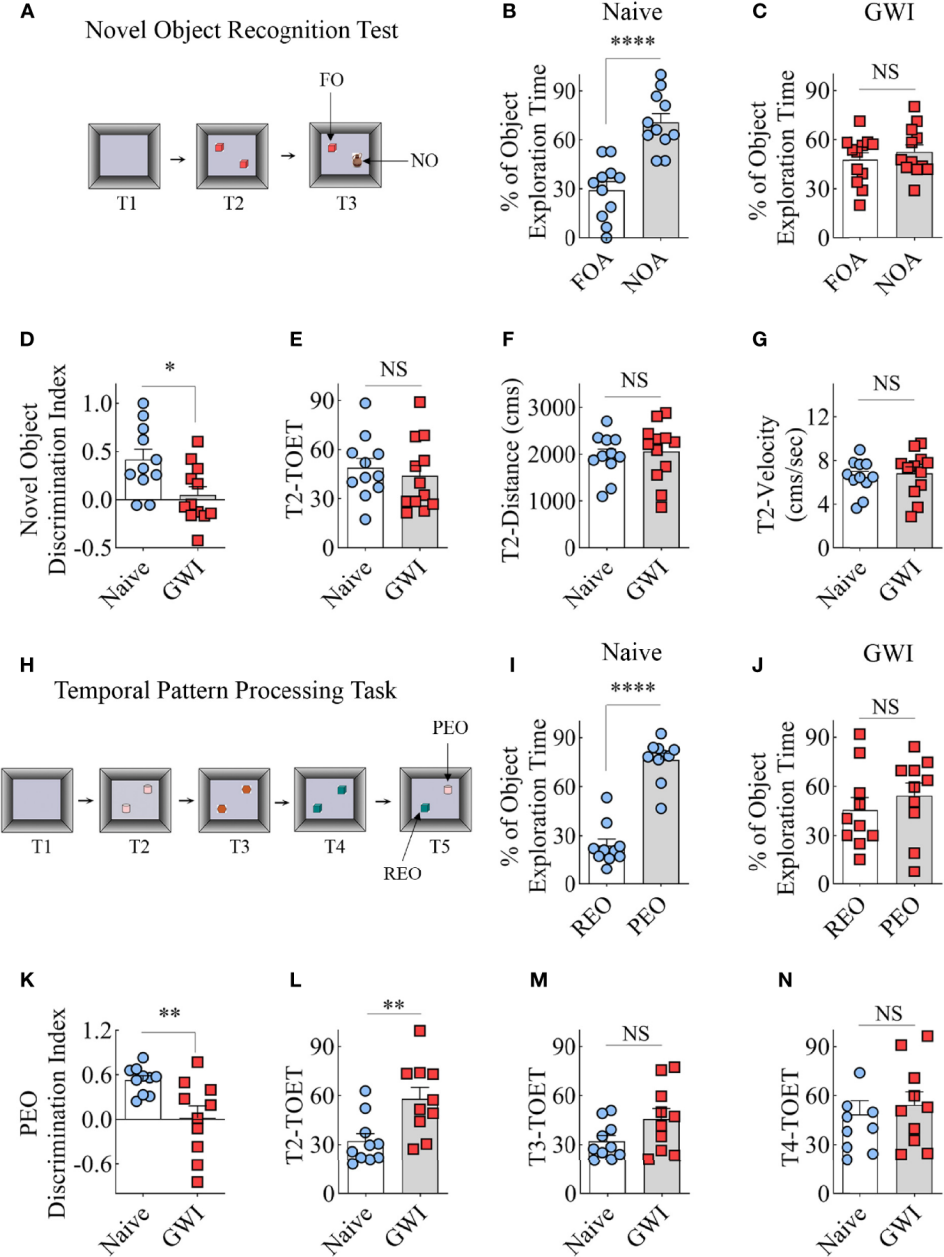
Animals with chronic Gulf War Illness (GWI) displayed recognition memory and temporal pattern processing impairments. Cartoon **(A)** illustrates different trials (T1-T3) involved in a novel object recognition test (NORT). The bar charts **(B, C)** compare percentages of time spent with the familiar object (FO) and the novel object (NO) in naïve **(B)** and GWI rats **(C)**. **(D)** compares the novel object discrimination index between naïve and GWI rats. The bar charts in **(D–G)** compare the total object exploration time (TOET, **E**), the distance moved **(F)**, and the velocity of movement **(G)** between the two groups in T2. The cartoon H depicts the sequence of trials (T1-T5) in the temporal pattern processing task (TPPT). The bar charts **(I, J)** compare the percentages of time spent with the recently explored object (REO) vis-à-vis the previously explored object (PEO) in naïve **(I)** and GWI rats **(J)**. **(K)** compares the PEO discrimination index between naïve and GWI rats. The bar charts in **(L–N)** compare TOETs between the two groups in T2 **(L)**, T3 **(M)**, and T4 **(N)**. *, p<0.05; **, p<0.01; ****, p<0.0001; NS, not significant.

The recognition memory function was assessed using a NORT (**Figure 1A**). Proficiency in this test depends mainly on the integrity of the perirhinal cortex and partly on the hippocampus. A predilection for exploring a novel object (NO) over a familiar object (FO) determines the animal’s proficiency in recognition memory in this test. The majority of animals (11-12 out of 13-14 examined) met the criteria set for the object exploration time, which is ≥16 seconds in T2 and ≥8 seconds in T3. Naive animals explored the NO for more extended periods than the FO (t=5.5, p<0.001, **Figure 1B**), which suggested competence for recognition memory function. On the other hand, GWI rats displayed impaired recognition memory, which was evident from exploring both FO and NO for nearly equal durations (t=0.8, p>0.05, **Figure 1C**). Analysis of the NO discrimination index further confirmed differences between the two groups (t=2.7, p<0.05, **Figure 1D**). The data such as TOETs, distance traversed, and velocities of the movement did not differ between the two groups (t=0.4-0.6, p>0.05, **Figures 1E**–**G**).

### Temporal Pattern Processing Ability Was Impaired in GWI Rats

The animals were next tested with a temporal pattern processing (TPP) task (**Figure 1H**). TPP task evaluated the competence of animals for recognizing and generating temporal patterns by encoding patterns through time. Such function depends mainly on the integrity of the hippocampal CA1 subfield (61, 62). In a temporal pattern, the order of the components in a sequence is the temporal order. Because the temporal pattern spans a specific duration, TPP requires a neural network capable of encoding short-term memories to maintain the entire component for a certain period. A memory for the order in which items/events have been experienced is the temporal order memory (63). It also involves distinguishing the relative recency of stimuli/events from preceding stimuli/events (64). Therefore, the animal needs to remember the temporal order (i.e., the order of the components in a sequence) after exploring three distinct pairs of identical objects (T2-T4) with an inter-trial interval of 60 minutes. In the final trial (T5), the animal’s proficiency in distinguishing a recently explored object (REO, i.e., one of the objects from T4) from a previously explored object (PEO, i.e., one of the objects from T2) was measured. The PEO is not a novel object as the memory of T2 is not extinguished but encoded as a short-term memory to maintain the overall component for the test duration. Animals with the capability for TPP prefer PEO over the REO due to their tendency to ignore recently explored objects. Overall, TPPT requires proficient short-term memory for the order in which items/events have been experienced.

Most animals (10 out of 12 in each group) met the total object exploration time criteria, which is ≥20 seconds in T2-T4 and ≥8 seconds in T5. Naïve control animals showed a high predilection to explore the PEO for extended periods than the REO in T5 (t=9.2, p<0.001, **Figure 1I**), implying capability for TPP. In contrast, GWI rats explored PEO and REO for nearly comparable amounts of time (t=0.8, p>0.05, **Figure 1J**), suggesting the incompetence for TPP. Differences between the naïve and GWI rats in TPP were also evident from the comparison of the PEO discrimination index (t=3.0, p<0.01, **Figure 1K**). The TOETs differed between the two groups in T2 (t=3, p<0.01, **Figure 1L**) but not in T3 and T4 (t=0.5-1.9, p>0.05, **Figure 1M, N**). Thus, a cognitive function linked to the hippocampal CA1 subfield is impaired in rats with chronic GWI.

### GWI Rats Displayed Increased 5-LOX Expression in Hippocampal and Cortical Neurons and Microglia

**Figure 2:**
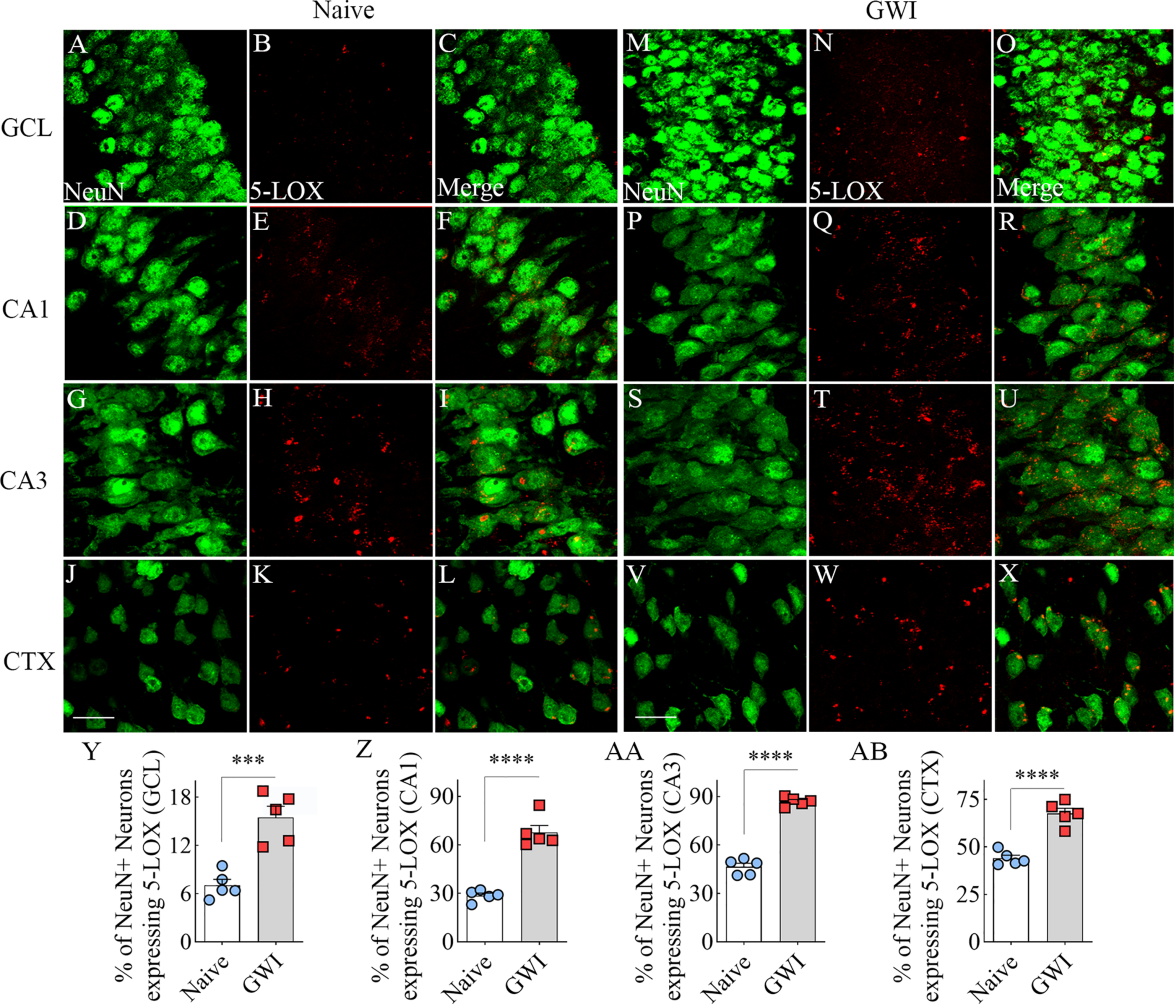
Neurons in the hippocampus and cerebral cortex displayed increased 5-lipoxygenase (5-LOX) expression in rats with chronic Gulf War Illness (GWI). Figures **(A–L)** illustrate 5-LOX expression (red) in NeuN+ neurons (green) from a naïve control rat in the granule cell layer (GCL, **A–C**), the hippocampal CA1 **(D–F)** and CA3 **(G–I)** subfields, and the cerebral cortex **(J–L)**. Figures **(M–X)** demonstrate 5-LOX expression (red) in NeuN+ neurons (green) from a rat with chronic GWI in the GCL **(M–O)**, the hippocampal CA1 **(P–R)**, and CA3 **(S–U)** subfields, and the cerebral cortex **(V–X)**. The bar charts **(Y-AB)** compare percentages of NeuN+ neurons expressing 5-LOX between naïve and GWI rats in the GCL **(Y)**, the hippocampal CA1 **(Z)**, and CA3 **(AA)** subfields, and the cerebral cortex **(AB)**. ***, p<0.001; ****, p<0.0001; Scale Bar = 25µm.

We first investigated the expression of 5-LOX, the synthesizing enzyme of LTs, in both hippocampal and cerebral cortical neurons from naïve control and GWI rats through dual immunofluorescence staining for NeuN and 5-LOX and Z-section analysis in a confocal microscope (**Figures 2A**–**X**). In comparison to NeuN+ neurons in naïve control animals, 5-LOX expression appeared considerably increased in NeuN+ neurons of GWI rats in different subfields of the hippocampus (**Figures 2A**–**U**) and the cerebral cortex (**Figures 2J**–**X**). Quantification revealed that higher percentages of NeuN+ neurons in GWI rats expressed 5-LOX in all hippocampal subfields and the cerebral cortex in comparison to their counterparts in naïve rats (t=5.4-16.3, p<0.001-0.0001, **Figure 2** **Y-AB**).

**Figure 3:**
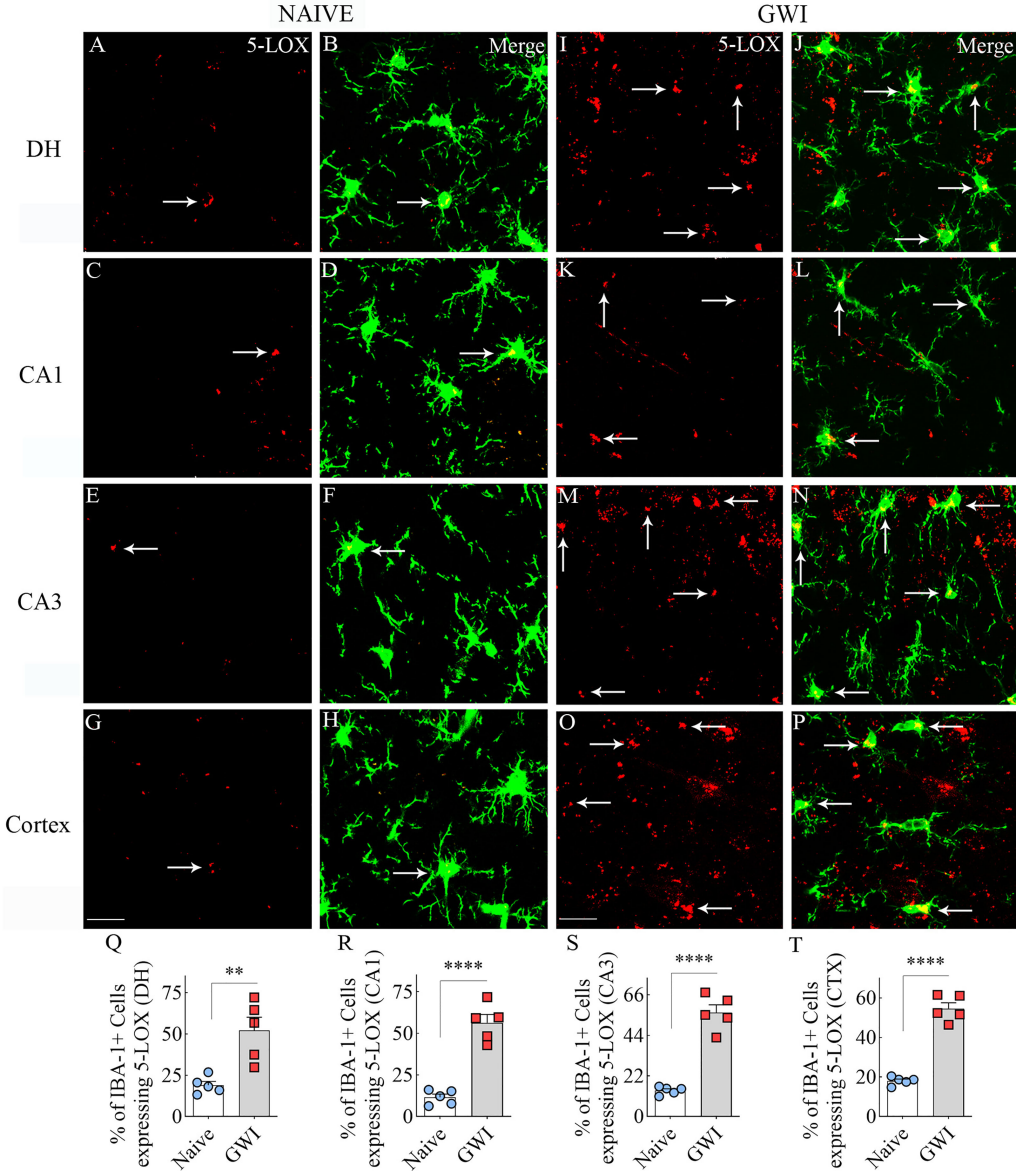
Microglia in the hippocampus and cerebral cortex displayed increased 5-lipoxygenase (5-LOX) expression in rats with chronic Gulf War Illness (GWI). Figures **(A–H)** illustrate 5-LOX expression (red) in IBA-1+ microglia (green) from a naïve control rat in the dentate hilus (DH, **A, B**), the hippocampal CA1 **(C, D)** and CA3 **(E, F)** subfields, and the cerebral cortex **(G, H)**. Figures **(I–P)** demonstrate 5-LOX expression (red) in IBA-1+ microglia (green) from a rat with chronic GWI in the GCL **(I, J)**, the hippocampal CA1 **(K, L)** and CA3 **(M, N)** subfields, and the cerebral cortex **(O, P)**. Arrows indicate 5-LOX expressing microglia. The bar charts **(Q–T)** compare percentages of IBA-1+ microglia expressing 5-LOX between naïve and GWI rats in the DH **(Q)**, the hippocampal CA1 **(R)** and CA3 **(S)** subfields, and the cerebral cortex **(T)**. **, p<0.01; ****, p<0.0001; Scale Bar = 25µm.

**Figure 4:**
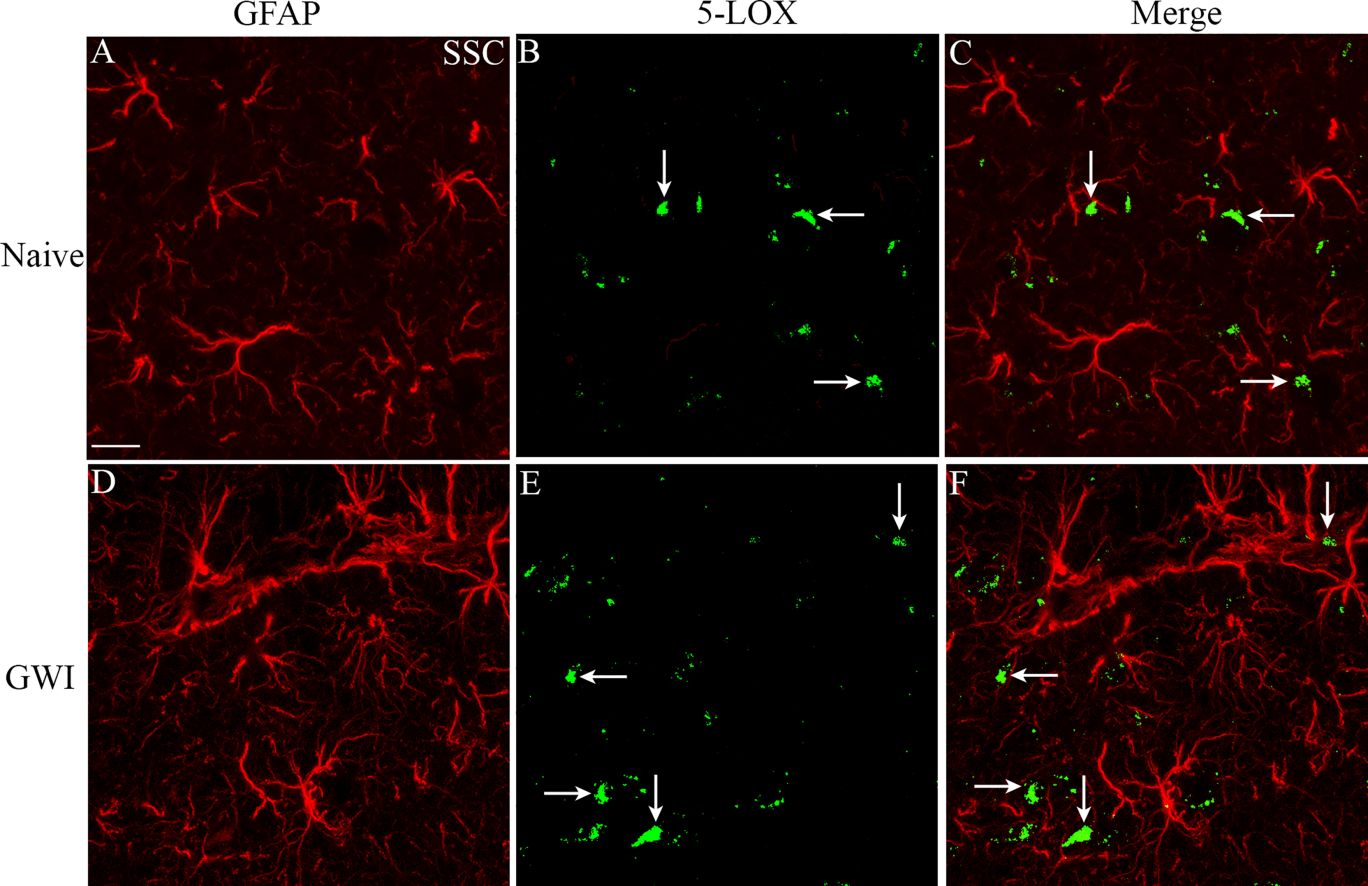
The soma of astrocytes (the area where multiple processes emanate) rarely displayed 5-LOX expression in both naïve control and GWI animals. Figures **(A–F)** illustrate representative examples of dual immunofluorescence staining for GFAP (red) and 5-LOX (green) in the cerebral cortex of a naive rat **(A–C)** and a GWI rat **(D–F)**. Arrows indicate a lack of 5-LOX expression in astrocytes. Scale Bar = 12.5 µm.

We next examined the expression of 5-LOX in both hippocampal and cerebral cortical microglia from naïve control and GWI rats through dual immunofluorescence staining for IBA-1 and 5-LOX and Z-section analysis in a confocal microscope (**Figures 3A**–**P**). 5-LOX expression appeared significantly increased in IBA-1+ microglia of GWI rats in different subfields of the hippocampus (**Figures 3A**–**N**) and the cerebral cortex (**Figures 3G**–**P**) compared to IBA-1+ microglia in naïve control rats. Quantification demonstrated that higher percentages of IBA-1+ microglia in GWI rats expressed 5-LOX in all hippocampal subfields and the cerebral cortex compared to IBA-1+ microglia in naïve rats (t=4.0-11.9, p<0.01-0.0001, **Figures 3Q**–**T**). We also evaluated whether 5-LOX is expressed in astrocytes through 5-LOX and GFAP dual immunofluorescence. Careful Z-section analysis in a confocal microscope revealed that the cell body region of astrocytes (the area where multiple processes emanate) rarely displayed 5-LOX expression in both naïve control and GWI animals. Because of this finding, we did not quantify the percentage of astrocytes expressing 5-LOX. Representative images of 5-LOX (green) and GFAP (red) are illustrated in **Figures 4A**–**F**.

### GWI Rats Displayed Higher Concentrations of 5-LOX, LTB4, and CysLTs in the Brain

**Figure 5:**
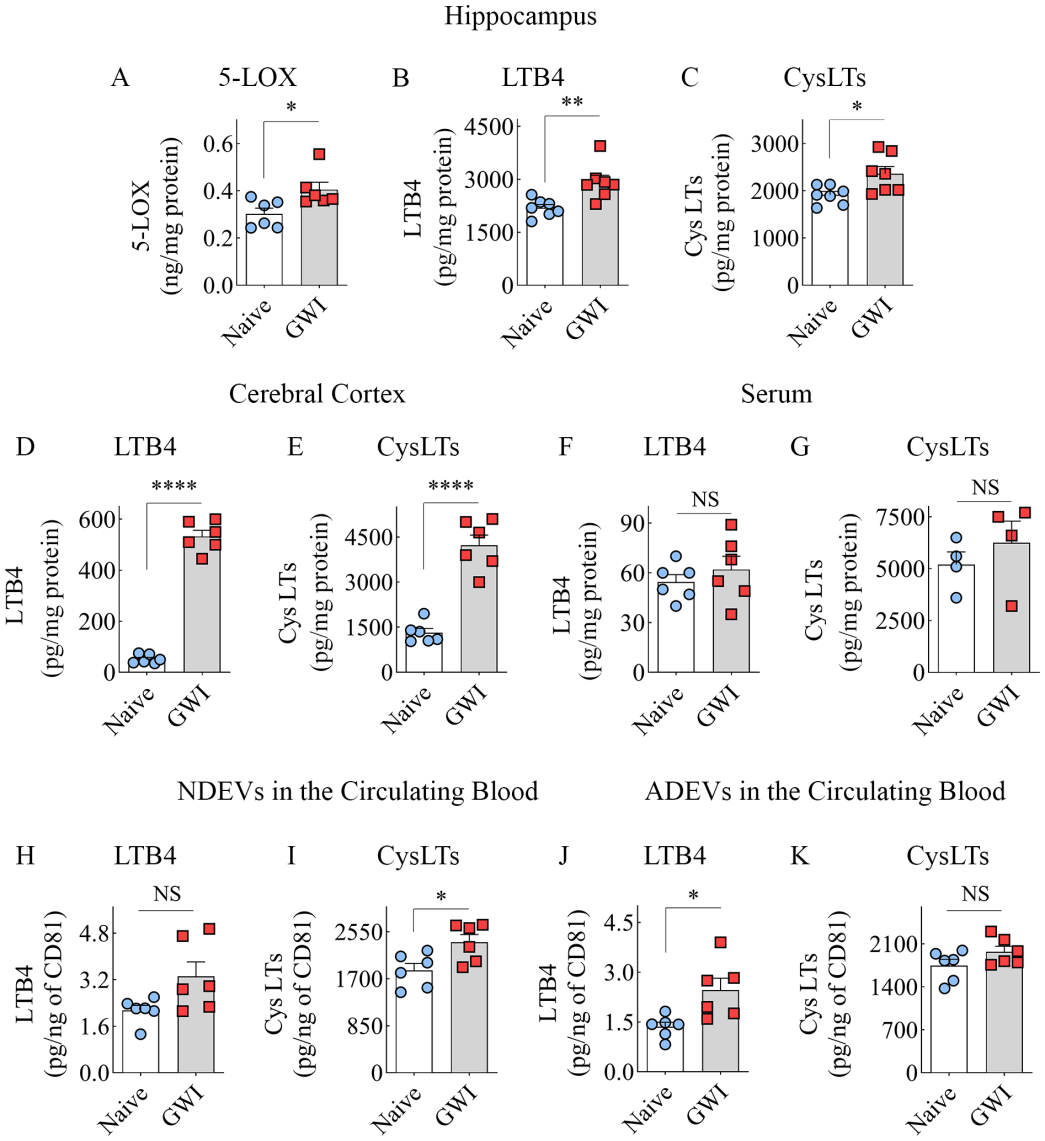
Animals with chronic Gulf War Illness (GWI) displayed an increased concentration of leukotrienes (LTs) in the brain and brain-derived extracellular vesicles (EVs) in the circulating blood but not in the serum. The bar charts **(A–C)** compare 5-LOX **(A)**, LTB4 **(B)**, and cysteinyl LT (CysLT; **C**) concentrations in the hippocampus, **(D, E)** compare LTB4 **(D)** and CysLTs **(E)** concentrations in the cerebral cortex, and **(F, G)** compare LTB4 and CysLT concentrations in the serum between naïve and GWI rats. The bar charts in **(H–K)** compare LTB4 and CysLT concentrations in neuron-derived extracellular vesicles (NDEVs **H, I**) and astrocyte derived EVs (ADEVs; **J, K**) between naïve and GWI rats. *, p < 0.05; **, p < 0.01; ****, p < 0.0001; NS, not significant.

We first measured 5-LOX concentration in hippocampal lysates from naïve and GWI rats to determine whether increased expression of 5-LOX in hippocampal neurons and microglia of GWI rats in the immunofluorescence study reflected a net increase in 5-LOX. Indeed, the hippocampus of GWI rats exhibited a significantly increased concentration of 5-LOX in comparison to the hippocampus of naïve rats (t=2.6, p<0.05, **Figure 5A**). Next, we examined whether increased 5-LOX levels in the hippocampus and the cerebral cortex resulted in higher production of LTs. Measurement through ELISA revealed that, in comparison to naïve rats, the GWI rats contained higher concentrations of LTB4 and CysLTs in the hippocampus and the cerebral cortex (t=2.7-19.2, p<0.05-0.0001, **Figures 5B**–**E**). Thus, GWI rats displayed increased LT signaling in the brain through increased expression of 5-LOX in both hippocampal and cerebral cortical neurons and microglia and increased concentration of LTB4 and CysLTs in the hippocampus and cerebral cortex.

### GWI rats did not display higher levels of LTB4 and CysLTs in the circulating blood

We probed whether an increased concentration of LTs observed in the brain is a global or brain-specific change in GWI by measuring LTB4 and CysLTs in the serum collected from the circulating blood. Notably, the concentrations of LTB4 or CysLTs in the serum were comparable between naïve control and GWI rats (t=0.8-0.9, p>0.05, **Figures 5F**, **G**), suggestive of no changes in LT signaling at the systemic level.

### Brain-derived EVs in the circulating blood contained higher levels of LTB4 and CysLTs

We examined whether increased LTB4 and CysLTs in the brain could be inferred by analyzing brain-derived EVs in the circulating blood by measuring the concentration of these LTs in NDEVs and ADEVs isolated from the blood. In comparison to NDEVs from naïve control rats, the NDEVs from GWI rats displayed no changes in LTB4 (U=7, p>0.05, **Figure 5H**) but contained higher levels of CysLTs (t=2.7, p<0.05, **Figure 5I**). Compared to ADEVs from naïve control rats, the ADEVs from GWI rats displayed higher concentration of LTB4 (t =2.9, p<0.05, **Figure 5J**) but no changes in CysLTs (t=1.7, p>0.05, **Figure 5K**). Thus, increased LTB4 concentration in the brain could be gleaned from LTB4 content in ADEVs, whereas elevated CysLTs in the brain could be deduced from CysLT concentration in NDEVs.

### GWI Rats Exhibited Increased Concentrations of Proinflammatory Cytokines in the Hippocampus, Cerebral Cortex and the Circulating Blood

**Figure 6:**
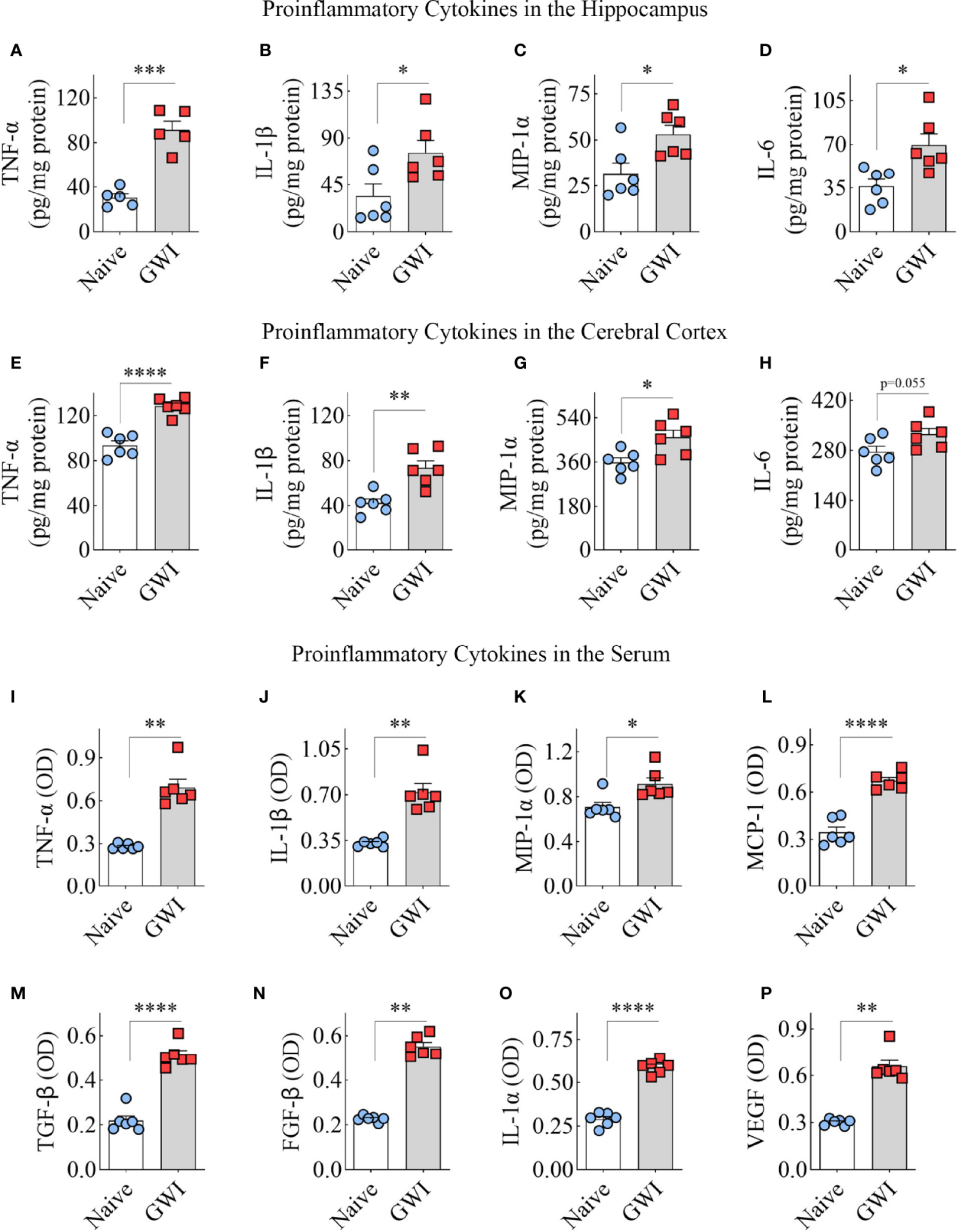
Animals with chronic Gulf War Illness (GWI) displayed an increased concentration of proinflammatory cytokines in the hippocampus, cerebral cortex, and circulating blood. The bar charts **A–H** compare tumor necrosis factor-alpha (TNF-α; **A, E**), interleukin-1 beta (IL-1β; **B, F**), macrophage inflammatory protein-1 alpha (MIP-1α; **C, G**), and interleukin 6 (IL-6; **D, H**) concentrations in the hippocampus **(A–D)** and the cerebral cortex **(E–H)** between naïve and GWI rats. The bar charts **(I–P)** compare TNF-α **(I)**, IL-1β **(J)**, MIP-1α **(K)**, monocyte chemoattractant protein-1 (MCP-1; **L**), transforming growth factor-beta (TGF-β; **M**), fibroblast growth factor-beta (FGF-β; **N**), interleukin-1 alpha (IL-1α; **O**), and vascular endothelial growth factor (VEGF; **P**) concentrations in the serum between naïve and GWI rats. *, p < 0.05; **, p < 0.01; ***, p < 0.001; ****, p < 0.0001.

To discern the extent of neuroinflammation and systemic inflammation in GWI rats, we measured the concentration of multiple proinflammatory cytokines in the hippocampus and the circulating blood. Compared to the hippocampus of age-matched naïve control rats, the hippocampus of GWI rats displayed higher levels of TNF-α, IL-1β, MIP-1α, and IL-6 (t=2.6-7.1, p<0.05-0.001, **Figures 6A**–**D**), revealing the presence of a chronic and robust neuroinflammatory environment in the hippocampus of GWI rats. Likewise, compared to the naïve control rats, the cerebral cortex of GWI rats revealed higher levels of TNF-α, IL-1β, and MIP-1α (t=3-6.9, p,0.05-0.0001, **Figures 6E**–**G**). The concentration of IL-6 was also higher in GWI rats than in naïve rats but did not reach statistical significance (t=2.2, p=0.055, **Figure 6H**). The serum collected from the circulating blood of GWI rats displayed concentrations of multiple proinflammatory proteins, including TNF-α, IL-1β, MIP-1α, monocyte chemoattractant protein-1 (MCP-1), transforming growth factor-β (TGF-β), fibroblast growth factor-β (FGF-β), IL-1α, and vascular endothelial growth factor (VEGF) (t/U=0.0-13.4, p<0.05-0.01, 0.0001, **Figures 6I**–**P**). Thus, rats afflicted with chronic GWI displayed an increased concentration of multiple proinflammatory proteins in the hippocampus and the circulating blood, implying concurrent neuroinflammation and systemic inflammation in GWI.

## Discussion

This study provides new evidence that chronic neuroinflammation and cognitive dysfunction in GWI are associated with increased LT signaling in the brain, and such an increase in LT signaling could be tracked through analysis of brain-derived EVs in the blood. Furthermore, a comparable blood concentration of LTs between age-matched naïve control and GWI rats revealed that altered LT signaling is not a global phenomenon but rather a brain-specific change in GWI.

A variety of cognitive tests have confirmed lasting cognitive impairments following exposure to GWI-related chemicals and stress in the rat model of GWI employed in this study (11, 12, 14–16, 18, 19, 49). In addition to confirming the recognition memory impairment reported earlier (12, 17), this study provides new evidence that temporal pattern processing task dependent on the hippocampal CA1 subfield is also impaired in chronic GWI. Cognitive impairments have also been observed in mouse models of GWI employing exposures to PB and PER (9, 20, 23, 33, 34) or organophosphate diisopropyl fluorophosphate (25). Moreover, neuroinflammation typified by the sustained activation of microglia and astrocytes has been recognized as one of the most conspicuous pathologies associated with cognitive impairments in both rat and mouse models of GWI (11, 15–17, 20, 23, 49, 65). Other studies have reported increased concentration of multiple proinflammatory cytokines in the brain in association with NLRP3 inflammasome activation in microglia (18, 19), leakage of HMGB1 into the extracellular space, and complement activation in the brain of rats with chronic GWI (16). Notably, positron emission tomography (PET) has validated neuroinflammation typified by activated microglia and astrocytes in veterans with chronic GWI (6). Thus, neuroinflammation is likely one of the major causes of persistent cognitive dysfunction in GWI because, depending on the severity, neuroinflammation can directly worsen cognitive function or indirectly affect cognition and mood by interfering with hippocampal neurogenesis (66, 67). Chronic neuroinflammation has been shown to degrade and reduce neural tissue and induce a leaky BBB, facilitating the entry of proinflammatory cytokines from the circulating blood (68, 69). However, specific mechanisms underlying the sustained activation of microglia and astrocytes after exposure to GWI-related chemicals are yet to be discovered.

An increased concentration of PC observed in the brain of a mouse model of GWI (34, 35) has suggested the possibility of elevated AA levels in the GWI brain as PC is the primary reservoir for AA. In such a scenario, proinflammatory LTs can also rise because AA is a precursor in the biosynthesis of LTs. Furthermore, the involvement of LTs in mediating BBB disruption and sustaining a chronic neuroinflammatory state has been well documented in neurodegenerative disorders, including AD (41, 44, 55, 70). The current study investigated whether neuroinflammation is linked with increased LT signaling in rats with chronic GWI. Indeed, our results showed increased levels of LTB4 and CysLTs in both hippocampus and the cerebral cortex of GWI rats, in association with increased concentrations of proinflammatory cytokines TNF-α, IL-1β, MIP-1α, and IL-6. Moreover, based on the increased expression of 5-LOX, the study showed that both neurons and microglia are the likely sources of increased LTs in GWI. However, based on studies in AD models, microglia are the major players in enhancing LT levels, as virtually all microglia express 5-LOX activating protein (FLAP) capable of anchoring 5-LOX to the membrane to mediate the contact with its substrate AA (55). Also, ablation of microglia in an AD mouse model reduced elements of the LT signaling pathway (55). However, it remains to be observed whether microglia are the sole mediators of increased LT signaling in GWI. Transient ablation of microglia using the colony-stimulating factor 1 receptor inhibitors may address this issue in the future.

LT-related studies have been primarily focused on their effects in conditions such as asthma and allergic disorders. However, the role of CysLTs as inflammatory mediators in neurodegenerative disorders has received significant attention lately because increased LT signaling can maintain a chronic neuroinflammatory state owing to several downstream effects. By acting on its receptors expressed on microvascular endothelial cells, CysLTs can cause endothelial cell dysfunction and BBB leakage (43, 71). CysLTs can also maintain a sustained activation of microglia and astrocytes through their receptors. Such effects have been observed in animal models of aging (72), and cerebral ischemia (39). The binding of CysLTs on microglial CysLT1 receptors can increase inflammatory response by elevating NF-kB signaling through mitogen-activated protein kinase, resulting in increased secretion of TNF-α and IL-1β (73). CysLTs have also been shown to interfere with the phagocytosis function of microglia and facilitate the production of free radicals by microglia in a model of Parkinson’s disease (45). Furthermore, studies have shown that restraining LT signaling in AD models can reduce amyloid-beta and tau loads and improve cognitive function (44, 47, 74–76). Also, restraining LT signaling in dementia patients induced beneficial effects on cognitive and mood function (77). From the above, the finding of increased LT signaling observed in the brain of rats with chronic GWI provides a new therapeutic target for GWI.

Another interesting finding in this study is that increased LT signaling in GWI rats is brain-specific, as the circulating blood did not exhibit elevated levels of LTB4 or CysLTs. Higher levels of LTB4 and CysLTs in brain-derived EVs in the circulating blood but not in the total serum further support the concept that increased LT signaling in GWI is brain-specific. The brain-specific alteration of LT signaling observed in this study implies that specific inflammatory mediators can get activated in the brain without its involvement at the systemic level. A similar pattern has been reported in a mouse model of GWI where exposure to corticosterone (CORT) and DFP induced neuroinflammation without causing systemic inflammation (22, 27, 78). However, the rat model of GWI employed in the study did exhibit chronic systemic inflammation in addition to neuroinflammation. The presence of systemic inflammation in GWI rats is evident from elevated levels of multiple proinflammatory proteins, including TNF-α, IL-1β, MIP-1α, MCP-1, TGF-β, FGF-β, IL-1α, and VEGF in the circulating blood. Earlier studies, including at earlier time points after exposure to GWI-related chemicals, have demonstrated such inflammation and elevated oxidative stress at the systemic level (14, 17). Collectively, the findings imply that mechanisms driving neuroinflammation and systemic inflammation are likely different in GWI. Previous studies in mouse models of GWI have suggested that systemic inflammation in GWI can result from adverse changes in the microbiota (79–81) and the persistence of haptenated pesticide metabolites (82). Regardless of the mechanisms, it is clear from this and previous studies that systemic inflammation parallels neuroinflammation in GWI. Systemic inflammation is likely an additive to neuroinflammation in mediating cognitive impairments, as proinflammatory cytokines from the circulating blood can enter the brain through a leaky BBB and exacerbate neuroinflammation.

## Conclusions and Future Studies

This study demonstrated that animals afflicted with chronic GWI showed an increased expression of 5-LOX and elevated levels of LTB4 and CysLTs in the brain, suggesting that enhanced LT-signaling is likely one of the mediators of persistent neuroinflammation in GWI. Furthermore, the study showed that changes in LT signaling in the brain could be tracked by analyzing brain-derived EVs in the blood, as reported earlier for other neuroinflammatory markers (16). The results also imply that increased LT signaling in the GWI brain can maintain BBB leakage (83) by inducing endothelial cell dysfunction and contraction of smooth muscle cells around small blood vessels. Persistently leaky BBB in GWI rats can allow blood-borne factors, particularly chronically increased levels of proinflammatory cytokines, including brain-function impairing cytokines such as TNF-α and IL-1β (14) to enter the brain and exacerbate brain dysfunction.

Nonetheless, the contribution of increased LT signaling in the brain to persistent cognitive dysfunction in GWI remains to be established. In this context, restraining LT signaling through interventional drugs such as montelukast, an antagonist of CysLTR1, or specific 5-LOX pathway antagonists (e.g., zileuton) would likely clarify whether enhanced LT signaling induced neuroinflammation underlies cognitive dysfunction in GWI. Both montelukast and zileuton can repair BBB through reduced LT signaling on endothelial cells and astrocytes and block LT-mediated inflammatory signaling cascade through astrocytes and microglia. Such effects are likely because montelukast can inhibit CysLT signaling at CysLTR1, and zileuton can reduce the synthesis of all LTs by inhibiting 5-LOX. By acting on CysLTRs, montelukast can invert leukotriene signaling, converting an inflammatory signal into an antiinflammatory signal (84). Modulating LT inflammatory signaling pathway in microglia and astrocytes by montelukast may provide a new avenue for overcoming brain dysfunction in GWI. Indeed, the beneficial effects of montelukast therapy have been seen in animal models of several human diseases, which include inhibition of microglial activation in a model of Parkinson’s disease (85), reduction of seizures in association with alleviation of oxidative stress and BBB dysfunction in models of epilepsy (43), and alleviation of memory impairment and neuroinflammatory responses in a model of AD (86). Thus, studies testing different doses of montelukast and/or zileuton in models of chronic GWI are needed in the future to ascertain the most efficient low doses that suppress chronic neuroinflammation and improve cognitive function without any adverse side effects. Moreover, since the current study was conducted on only male animals, future studies need to examine whether a similar brain-specific increase in LT signaling occurs in female animals with chronic GWI.

## Data Availability Statement

The raw data supporting the conclusions of this article will be made available by the authors, without undue reservation.

## Ethics Statement

The animal study was reviewed and approved by IACUC of the Texas A&M University.

## Author Contributions

Concept: AKS. Research design and data collection: SA, RU, MK, LNM, DU, BS, and AKS. Data analysis and interpretation: SA, RU, MK, LNM, DU, and AKS. The first draft of the manuscript text: SA and RU. Draft Figures: SA, RU, and MK. Finalization of manuscript text and figures: AKS. All authors provided feedback and approved the final version of the manuscript.

## Funding

This work was supported by the Department of Defense Gulf War Illness Research Program grants, W81XWH-16-1-0480, W81XWH-17-1-0447, and W81XWH-19-1-0548 to AS.

## Author Disclaimer

The contents of this article suggest the views of the authors and do not represent the views of the Department of Defense or the United States Government.

## Conflict of Interest

The authors declare that the research was conducted in the absence of any commercial or financial relationships that could be construed as a potential conflict of interest.

## Publisher’s Note

All claims expressed in this article are solely those of the authors and do not necessarily represent those of their affiliated organizations, or those of the publisher, the editors and the reviewers. Any product that may be evaluated in this article, or claim that may be made by its manufacturer, is not guaranteed or endorsed by the publisher.
