# Corrigendum: Brain-specific increase in leukotriene signaling accompanies chronic neuroinflammation and cognitive impairment in a model of Gulf War Illness

**DOI:** 10.3389/fimmu.2025.1557065

**Published:** 2025-01-31

**Authors:** Sahithi Attaluri, Raghavendra Upadhya, Maheedhar Kodali, Leelavathi N. Madhu, Dinesh Upadhya, Bing Shuai, Ashok K. Shetty

**Affiliations:** Institute for Regenerative Medicine, Department of Molecular and Cellular Medicine, College of Medicine, Texas A&M University Health Science Center, College, Station, TX, United States

**Keywords:** cysteinyl leukotrienes, gulf war illness (GWI), gulf war-related chemicals, cytokines, 5-lipoxygenase, neuroinflammation, leukotriene signaling, blood-brain barrier

In the published article, there was an error in **Figures 5B**–**G** as published. In **Figure 5**, the bar charts B, C, D, E, F, G, y-axis labels were inadvertently misspelled as “ng/mg protein”, instead of “pg/mg protein.”. The corrected **Figure 5** and its caption appear below.

**Figure 5:**
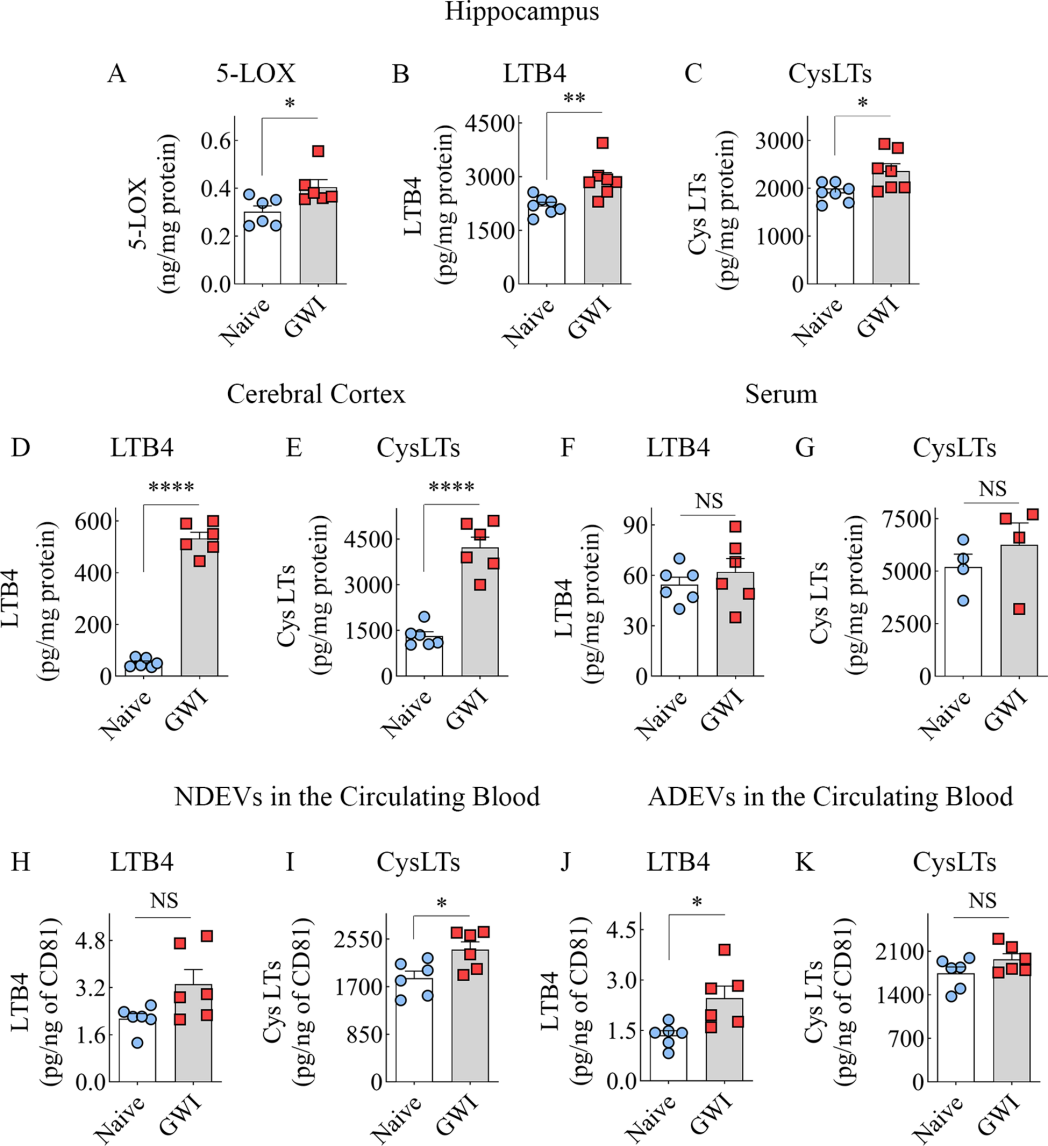
Animals with chronic Gulf War Illness (GWI) displayed an increased concentration of leukotrienes (LTs) in the brain and brain-derived extracellular vesicles (EVs) in the circulating blood but not in the serum. The bar charts **(A–C)** compare 5-LOX **(A)**, LTB4 **(B)**, and cysteinyl LT (CysLT; **C**) concentrations in the hippocampus, **(D, E)** compare LTB4 **(D)** and CysLTs **(E)** concentrations in the cerebral cortex, and **(F, G)** compare LTB4 and CysLT concentrations in the serum between naïve and GWI rats. The bar charts in **(H–K)** compare LTB4 and CysLT concentrations in neuron-derived extracellular vesicles (NDEVs **H, I**) and astrocyte derived EVs (ADEVs; **J, K**) between naïve and GWI rats. *, p < 0.05; **, p < 0.01; ****, p < 0.0001; NS, not significant.

The authors apologize for this error, and this does not change the scientific conclusions of the article in any way. The original article has been updated.

